# Editorial: Novel biomarkers for early diagnosis involved in autoimmune and autoinflammatory diseases

**DOI:** 10.3389/fimmu.2026.1810163

**Published:** 2026-02-20

**Authors:** Jan Stępniak, Katarzyna Wojciechowska-Durczyńska, Małgorzata Karbownik-Lewińska

**Affiliations:** Department of Endocrinology and Metabolic Diseases, Medical University of Lodz, Lodz, Poland

**Keywords:** autoimmune diagnostics, autoimmune diseases, autoinflammatory diseases, early diagnosis, innate and adaptive immunity, management in autoimmunity, novel biomarkers

Autoimmune and autoinflammatory diseases affect a significant portion of the global population, imposing a substantial burden on healthcare systems and reducing patients’ quality of life. These disorders are characterized by heterogeneous clinical presentations and overlapping symptoms, which complicate diagnosis and long-term management. Identifying specific and sensitive biomarkers is essential for improving patient outcomes. In particular, utilizing the “window of opportunity” for early intervention, a period during which treatment can fundamentally alter disease progression, requires a transition from traditional clinical observation to molecular and systems-based diagnostics.

This Research Topic, “Novel Biomarkers for Early Diagnosis Involved in Autoimmune and Autoinflammatory Diseases,” showcases research that integrates molecular biology, clinical immunology, and bioinformatics to advance diagnostic innovation and clarify disease mechanisms.

Several contributions have highlighted the role of novel biomarkers derived from routine blood counts as accessible and cost-effective markers of systemic inflammation. Liang et al. and Liu et al. revealed that the neutrophil-to-HDL ratio (NHR) and the neutrophil-percentage-to-albumin ratio (NPAR) are independently associated with psoriasis risk and rheumatoid arthritis (RA) prevalence, respectively. Similarly, Yu et al. demonstrated the diagnostic and prognostic value of biomarkers such as the neutrophil-to-lymphocyte ratio (NLR), the platelet-to-lymphocyte ratio (PLR), the monocyte-to-lymphocyte ratio (MLR), the systemic immune-inflammation index (SII), and the systemic inflammation response index (SIRI) in ANCA-associated vasculitis, emphasizing their potential utility in monitoring disease activity and predicting renal involvement.

At the molecular and transcriptomic levels, researchers have utilized bioinformatics and machine learning to identify novel drivers of disease. Zheng et al. identified cytoskeleton-associated protein 2 (CKAP2) as a novel hub gene in the RA synovium; their study demonstrates that CKAP2 promotes disease progression by stimulating the proliferation, migration, and invasion of fibroblast-like synoviocytes (FLS), which are fundamental to synovial hyperplasia and joint destruction. Feng et al. linked a signature of five telomere-related genes (ABCC4, S100A8, VAMP2, PIM2, and ISG20) to RA pathogenesis; these diagnostic markers correlate with cell cycle pathways and, based on molecular docking, represent potential therapeutic targets for drugs such as methotrexate. Applying these advanced analytics to skin disorders, Wang et al. identified HMGA1 and PSMD13 as vitiligo biomarkers using WGCNA and machine learning. Through blood-based qRT-PCR and skin transcriptomic analysis, they demonstrated that PSMD13 drives lesion development via the Nod-like receptor signaling pathway, whereas HMGA1 acts primarily as a circulating marker, providing novel targets for diagnostic and therapeutic innovation.

The genetic architecture of susceptibility and disease course is further explored through specific molecular variants. Kril et al. described how ERAP1/2 polymorphisms distinguish RA patients from healthy controls and influence clinical phenotypes, highlighting serum ERAP2 protein as a valuable biomarker of disease severity. Gabryel et al. identified a rare CXCL8 (IL-8) stop-gained variant (c.91T), which is significantly more frequent in inflammatory bowel disease (IBD) patients and is associated with aggressive disease courses requiring corticosteroids or surgery. Their findings suggest that this variant may influence therapy response by modulating the complex, concentration-dependent role of IL-8 in neutrophil motility, marking it as a potential pharmacogenetic target. Complementing these findings, Tian et al. defined NAD^+^ metabolism–related subtypes in ulcerative colitis and identified diagnostic biomarkers, including NNMT, PARP9, and NAMPT, utilizing them to develop accurate nomograms for disease subtyping and clinical management.

The challenge of diagnostic standardization and technical innovation is a recurring theme. Appeltshauser et al. provided a crucial inter-laboratory comparison for FGFR3 antibody testing in sensory neuropathies, establishing and evaluating a detailed ELISA protocol for a robust diagnostic assessment. In the realm of advanced proteomics, Burbelo et al. introduced a luminescent autoantibody profiling system (LIPS) to accurately quantify a broad spectrum of myositis- and lung-related autoantibodies in patients with idiopathic inflammatory myopathies (IIM) and Sjögren’s disease. By overcoming the limitations of standard assays, this high-dynamic-range platform identifies markers of interstitial lung disease (ILD) activity, facilitating the precise characterization of clinically relevant patient subsets and treatment response. Furthermore, Yamaka et al. reported that decreased serum sulfatide levels serve as a specific metabolic marker reflecting the pathological activity of lupus nephritis.

Cross-disease and multi-modal approaches have also yielded significant insights. Li et al. identified five shared transcriptomic biomarkers (KIF4A, DLGAP5, NCAPG, CCNB1, and CEP55) between psoriasis and Crohn’s disease, revealing shared pathogenic mechanisms involving cell cycle regulation and T-cell-mediated inflammation. Furthermore, their study suggests drug repurposing opportunities, identifying Etoposide and Piroxicam as potential treatments targeting these hubs. In the search for high-resolution diagnostics, Petzinna et al. presented an exploratory case report representing the first application of [68Ga]Ga-DOTA-Siglec-9 PET/CT in the context of Giant Cell Arteritis (GCA) relapse. By targeting Vascular Adhesion Protein-1 (VAP-1), this novel method allows for the precise localization of systemic vascular inflammation, potentially complementing established imaging techniques to address the limitations of traditional diagnostics and enhance therapeutic management. For complex overlapping phenotypes, Gan et al. demonstrated the clinical utility of the random forest algorithm in distinguishing RA-SS from SS-PA. Their model prioritizes parameters such as anti-CCP levels, RF levels, and erosive joint count to provide clinicians with enhanced diagnostic criteria, aiming to bridge the gap between early detection and improved long-term patient outcomes.

Finally, this Research Topic has addressed rare phenotypes and experimental models. Bizjak et al. provided a comprehensive characterization of cold-induced anaphylaxis, demonstrating that a higher prevalence of HαT and KIT p.D816V contributes to its pathogenesis. The study highlights the diagnostic utility of assessing basal serum tryptase to identify these variants and proposes elevated IgE as a potential biomarker specific to cold-induced anaphylaxis. From a translational perspective, Kakan et al. used Sjögren’s disease mouse models to demonstrate that autoimmune dacryoadenitis produces distinct tear autoantibody profiles, with IgA showing greater diversity than IgG. The authors identified tertiary lymphoid structures (TLS) in the lacrimal gland as the likely source of these antibodies, indicating that tear composition mirrors localized glandular pathology more closely than that of serum.

Collectively, the studies in this Research Topic demonstrate how translational immunology can shift disease classification from clinical symptoms to underlying molecular drivers. Utilizing advanced analytics and molecular technologies, these studies provide a framework for more precise diagnostics. Future progress depends on validating these experimental and computational findings in large prospective cohorts and on integrating multi-omics data into clinical practice. Standardizing protocols and fostering international collaboration remain essential for incorporating these biomarkers into routine precision medicine. We expect that the insights presented in this Research Topic will support the development of biomarker-driven diagnostics and improve the accuracy of early interventions in autoimmune and autoinflammatory disorders.

